# The effect of sex of last child on short birth interval practice: the case of northern Ethiopian pregnant women

**DOI:** 10.1186/s13104-019-4110-x

**Published:** 2019-02-04

**Authors:** Amare Genetu Ejigu, Ayenew Engida Yismaw, Miteku Andualem Limenih

**Affiliations:** 1 Department of Midwifery, College of Health Science, Mizantepi University, Mizantepi, Ethiopia; 2 Department of Midwifery, College of Medicine and Health Sciences, University of Gondar, Gondar, Ethiopia

**Keywords:** Birth interval, Pregnant women, Ethiopia, Short birth interval practice

## Abstract

**Objective:**

Improving short birth interval practice is a key strategy to reduce maternal mortality, neonatal mortality, adverse pregnancy outcomes, high fertility rate and undermining economic development efforts. However, there were limited evidences on short birth interval practice and its determinant factors in Ethiopia. This study aimed to determine the prevalence of short birth interval practice and associated factors among pregnant women. Institutional based cross-sectional study was conducted among 418 pregnant mothers using stratified sampling technique. Multivariable logistic regression analysis was performed at the level of significance of *P*-value < 0.05.

**Result:**

Short birth interval practice was found to be 40.9%. Child death (AOR = 3.60, 95% CI 1.35, 9.59), female child (AOR = 2.03, 95% CI 1.12, 3.67), younger maternal age (AOR = 4.23, 95% CI 1.14, 12.66), contraceptive non-use (AOR = 8.15, 95% CI 4.17, 15.94), increase duration of breastfeeding (AOR = 4.72, 95 CI% 1.10, 20.60) and home delivery (AOR = 4.75, 95 CI% 2.30, 9.79) were found to be significantly associated with short birth interval practice. The prevalence of short birth interval practice is high. Multi disciplinary approach through improving maternal and child health care are recommended to prevent short birth interval practice.

## Introduction

Birth interval is the length of time between two successive live births [1]. Evidences showed that birth intervals should be spaced for three to 5 years apart to ensure maximum health benefits for mothers and newborns, which is recommended by WHO; after a live birth, the interval before attempting the next pregnancy is at least 24 months [2–4].

Globally, there were 287, 000 maternal deaths and 3.1 million neonatal deaths in 2013 and most of these occurred in south Asia and in sub-Saharan Africa [5, 6]. EDHS 2016 showed; there is high maternal mortality, 412 deaths per 100,000 live births [7] and more than 42% of under-5 mortality was neonatal deaths [8]. Short birth interval increase risk of maternal mortality and adverse pregnancy outcomes (neonatal mortality, low birth weight, small fetus, and pre-term delivery) [3, 9].

Studies done in USA, DHS from 66 low and middle-income countries and in Bangladesh showed; neonatal, infant, child mortality and stillbirth are high when the women practice short birth interval [10–12]. Evidences showed that, birth interval less than 18 months leads to 12% neonatal death, 84.4% LBW and decrease neonatal survival [13–16].

In Ethiopia, Short birth interval affects fertility as well as neonatal, infant and Childhood mortality [17]. Promoting the length of birth interval for a minimum of two years results reduction of infant mortality by 50% and reduces fertility rate by 43% [18–20].

Beyond the health implications, close birth intervals increase population growth, makes women to be less productive and the family will invest more of their limited resources to child care [2].

According to researches; maternal age, level of education, marital status, residence, loss of child, sex of child, contraceptive use, breast feeding, and age of pregnancy are contributing factors [1, 21–28]. However, the determinants of short birth interval practice are not the same across different cultures and socio-demographic status within the society. Therefore, this study aimed to assess factors affecting short birth interval practice among pregnant women in Debremarkos town governmental health institutions, Ethiopia.

## Main text

### Study setting

The study was conducted at Debremarkos governmental health institutions from April 1 to July 30, 2016. The town has 101,582 populations [29]. The numbers of governmental health institutions in the town were four. 10 BSc, 14 diploma Midwives and 8 diploma Nurses were providing health care services in the maternal care unit of these health institutions. The study was done using institutional based cross-sectional design on all pregnant women who had at least one live birth during ANC visit in debremarkos town governmental health institutions during the study period.

### Required sample size

Single population proportion formula was used to calculate the total required sample size of the study considering the assumption of 95% confidence level, 5% margin of error, 57.6%(P) the proportion of short birth interval practice which is taken from previous study [24] and considering 10% of possible non response rate. This gave us the total required sample size of the study to be 418.

### Sampling technique

Systematic random sampling techniques were used to select the required sample size then through distributing the required sample size to each governmental health institution allocated in the town based on proportional number of multigravida pregnant women who had ANC visit during study period. For procedure, we used sampling interval approach. Sampling interval (K) was calculated using the summation of 4 months multigravida pregnant women who had ANC visit in all governmental health institutions, which were 920. Then K = N/n, 920/418 = 2.201 ≈ 2. Every 2nd pregnant women were interviewed. To start with the first interview we used lottery method.

### Operational definition

Short birth interval: if the interval between last birth and current pregnancy expected date of delivery is < 33 months.

### Data collection techniques

The data was collected using Amharic questionnaire which is translated from English language. Before the actual data collection; pre-test was done on 5% of total sample size out of the study area which has similar socio demographic context. To collect all the required data we used four diploma midwives.

### Data analysis techniques

SPSS version 20 software was used to analyze the data. Variables having P-value of < 0.20 in bivariate analysis were entered into multiple logistic regressions for further analysis. Finally, variables with P-value < 0.05 in multivariable logistic analysis were considered as statistically significant.

### Socio-demographic Characteristics of the Participants

A total of 418 pregnant women who came for ANC follow-up in Debremarkos governmental health institutions were interviewed with a response rate of 98.3%. The mean age was 30 years (SD ± 5.14), 405 (96.1%) mothers were Orthodox Christians, 261 (63.5%) mothers lived in urban area (Table 1).

**Table 1:** Socio-demographic characteristics of multi gravid pregnant women who had ANC follow-up in Debremarkos town governmental health institution, North West Ethiopia, 2016 (n = 411)

Variables	Categories	Frequency (n = 411)	Percent (%)
Residence	Urban	261	63.5
	Rural	150	36.5
Religion	Orthodox	395	96.1
	Muslim	11	2.7
	Protestant	4	1.0
	Catholic	1	0.2
Marital status	Married	401	97.6
	Widowed	1	0.2
	Divorced	9	2.2
Educational status	No education	173	42.1
	Primary (1–8)	106	25.8
	Secondary and above	132	32.1
Occupational status	Government Employee	71	17.3
	House wife	253	61.6
	Merchant	60	14.6
	Student	27	6.6
Husband education (n = 401)	No education	110	27.4
	Primary (1–8)	119	29.7
	Secondary and above	172	42.9
Husband occupation (n = 401)	Government Employee	132	32.9
	Farmer	137	34.2
	Merchant	99	24.7
	Daily laborer	33	8.2
Ethnicity	Amhara	388	94.4
	Oromo	14	3.4
	Tigre	6	1.5
	Gurage	3	0.7
Age	15–24	47	11.4
	25–29	151	36.7
	30–34	115	28.0
	35–39	74	18.0
	40–49	24	5.8

### Child related characteristics

Two hundred sixty-three (63.9%) mothers had 1 or 2 alive children, 96 (23.4) had 3 or 4 alive birth and 52 (12.7%) had ≥ 5 alive children. One hundred fifty-one (36.7%) and 139 (33.8%) women had no alive male and female child respectively. Two hundred four (49.6%) mother`s last child were male. Eighty-eight (21.4%) mother`s last child were not alive. 385 (96.7%) mothers had designer for more child. Among these; 236 (63.1%) mothers needs 1 to 2 more children, 120 (32.1%) mothers needs 3–4 more children and 18 (4.8%) mothers needs more than 4 children. 100 women`s (24.3%) current pregnancy was unplanned.

### Maternal and reproductive related characteristics

Two hundred eighty (68.1%) of the respondent gave their last live birth in the age group of 20–29 years. 352 (85.6%) women had breastfed their last child. 242 (59%) pregnant women had breastfed their last child for 24 months and more. 357 (86.9%) respondents were ever used contraceptives and 257 (72%) were used contraceptives before this pregnancy. Nearly one-third (32.4%) of study participants gave their last live birth at home. Forty-two (10.2%) respondents gave their last birth through cesarean Sect. 243 (59%), 66 (16%), 25 (6%) and 78 (19%) mothers had breastfed their last child for ≥ 24, 13–23, 7–12 and 1–6 months respectively.

### Birth interval practice

**Fig. 1:**
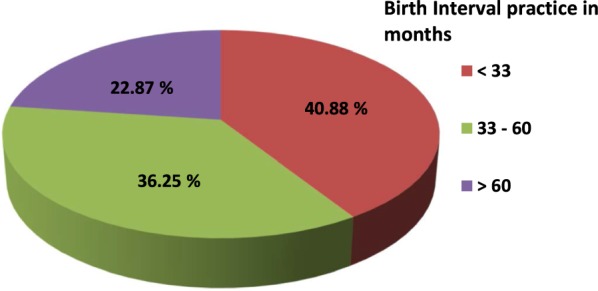
Distribution of birth interval practice among pregnant women who has ANC follow-up in Debremarkos town governmental health institution, Northwest Ethiopia, 2016 (n = 411)

One hundred sixty-eight (40.9%) of the study participants had practiced short birth interval. The median duration of birth interval practice was 37 months (SD ± 16.7) (Fig. 1).

### Factors associated with short birth interval practice

Sex of last child, age of the mother, survival status of last child, duration of breast feeding before conception of the current pregnancy, contraceptive use and place of birth of last child were significant factors for short birth interval practice. The odds of pregnant mothers whose last child were female were 2 times more likely to have short birth interval practice (AOR = 2.03, 95% CI 1.12, 3.67). Those pregnant women who were in the age group of 15–24 years had 4 times more likely to have short birth interval practice as compared to those who are in the age group of 25–29 (AOR = 4.23, 95% CI 1.14, 12.66) and women who were in the age group 35–39 years and 40–49 years had 27 and 23 times less likely to have short birth interval practice as compared to those women who were at the age of 25–29 years (AOR = 0.037, 95% CI 0.004, 0.37 and AOR = 0.044, 95% CI 0.003, 0.62) respectively. Women whose last child died were 3.6 times more likely to have short birth interval practice as compared with women whose child were alive (AOR = 3.60, 95% CI 1.35, 9.59). Women who breastfed their last child for 13–23 months were 4.7 times more likely to have short birth interval as compared to those who breastfed for < 6 months (AOR = 4.72, 95 CI% 1.10, 20.60). Mothers who were not using contraceptives between their last birth and this pregnancy had 8 times more likely to have short birth interval as compared to those women who used it (AOR = 8.15, 95% CI 4.17, 15.94). Mothers who gave their last birth at home were 4.7 times more likely to have short birth interval as compared to women who gave birth at health institution (AOR = 4.75, 95 CI% 2.30, 9.79) (Table 2).

**Table 2:** Logistic regression showing factors on short birth interval practice among mothers who came for ANC clinic in Debremarkos governmental health institution, Ethiopia, 2016 (n = 411)

Variables	Categories	Short birth interval		COR (95% CI)	AOR (95% CI)	P-value
		Yes	No			
Age of mother	15–24	27	20	2.29 (1.18, 4.46)	*4.23 (1.14, 12.66)*	0.010
	25–29	56	95	1	1	
	30–34	45	70	1.09 (0.66, 1.79)	0.73 (0.34, 1.55)	0.408
	35–39	29	45	1.09 (0.62, 1.94)	*0.037 (0.004, 0.37)*	0.005
	40–49	11	13	1.44 (0.60, 3.42)	*0.044 (0.003, 0.62)*	0.021
Sex of last child	Male	72	132	1	1	
	Female	96	111	1.58 (1.07, 2.36)	*2.03 (1.12, 3.67)*	0.020
Last child alive	Yes	118	206	1	1	
	No	50	37	2.36 (1.46, 3.82)	*3.60 (1.35, 9.59)*	0.010
Duration of breast feeding	0–6	44	37	1	1	
	7–12	15	9	1.40 (0.55, 3.57)	2.53 (0.46, 13.82)	0.283
	13–23	45	20	1.89 (0.95, 3.75)	*4.72 (1.10, 20.60)*	0.039
	≥ 24	64	177	0.30 (0.18, 0.51)	0.87 (0.23, 3.24)	0.837
Contraceptive use	Yes	69	188	1	1	
	No	69	31	6.06 (3.66, 10.05	*8.15 (4.17, 15.94)*	0.000
Last child place of delivery	Health institution	101	177	1	*1*	
	Home	67	66	1.78 (1.17, 2.70)	*4.75 (2.30, 9.79)*	0.000

P < 0.05 Statistically significantly associated with short birth interval practice in multivariable logistic regression analysis

P ≥ 0.05 is not significant in stepwise backward logistic regression. Hosmer and Lemanshow test for multivariable log reg. = 0.85

The italicized value indicated that a statistically significant association at 95% confidence interval (CI) that did not include 1 in the interval

1 = reference category

### Discussion

This study revealed that short birth interval practice was found to be 40.9% (95% CI 35.9, 46.2). This finding is in line with EDHS report, 41.5% [1]. However, it is remarkably lower than the studies done in Tanzania (48.4%) and Southern Ethiopia (57.6%) [23, 24]. This might be the difference in socio demographic characteristics and study period variation. The difference in prevalence across studies might be socio demographic feature of the study participants, the time gaps between the study periods, accessibility of contraceptive methods and the difference in data collection methods [2, 14, 16].

Pregnant mothers whose last child died were 3.6 times more likely to have short birth interval than who had live child. (AOR = 3.60, 95% CI 1.35, 9.59). This finding is in line with studies done in northwest Ethiopia and in Bangladesh [12, 26, 27]. This might be due to socio- cultural influence and lack of access for contraceptive methods. When the child dies, the woman is less likely to be protected from pregnancy, socio-cultural influences hinders to go to health institution early for contraceptive methods and there is a high need of replacing the dead child soon with new pregnancy [2, 28].

Women who were in the age group 15–24 were 4 times (AOR = 4.23, 95% CI 1.14, 12.66) more likely to have short birth interval compared to 25–29 age group. Women who were in the age group 35–39 and 40–49 had 27 times (AOR = 037, 95% CI 0.004, 0.37) and 23 times (AOR = 0.044, 95% CI 0.003, 0.62) less likely to have short birth interval as compared to those age 25–29. This is in consistent with the finding of EDHS, southern Ethiopia and Tanzania [1, 23, 25]. This could be older women are more likely to have the desired number of children and as the woman’s age increases, fecundity decreases [6, 20].

Mothers who were not utilized contraceptive methods between their last birth and this pregnancy were 8 times more likely to practiced short birth interval as compared to users (AOR = 8.15, 95% CI 4.17, 15.94). This finding is in line with study done in southern Ethiopia [24, 25]. This might be due to contraceptive utilization can delay the pregnancy. When the women use contraceptive methods, they are intentionally preventing short birth interval and its bad outcomes [2, 3].

Pregnant mothers whose last child were female had 2 times more likely to have short birth interval as compared to mothers who had male last child (AOR = 2.03, 95% CI 1.12, 3.67). This finding is consistent with study done in southern Ethiopia and in Saudi Arabia [25, 28]. This might happen due to socio-cultural influence.

Those women who were breastfed for 13–23 months had 4.7 times more likely to have short birth interval as compared to those who breastfed for < 6 months (AOR = 4.72, 95 CI% 1.10, 20.60). This finding is in line with study done in southern Ethiopia [24].

Women might believe in, breast feeding can prevent pregnancy for long period of time and they may not use contraceptive methods which might expose them to be pregnant [22].

Mothers who gave their last birth at home were 4.75 times more likely to have short birth interval as compare to women who gave birth at health institution (AOR = 4.75, 95 CI% 2.30, 9.79). This is in line with the study conducted in Tanzania and Dabat district [23, 26]. This might be due to women who gave birth in the health facilities had more chance to have awareness through effective counseling and may receive post partum family planning methods [30].

### Conclusions and recommendation

Short birth interval practice was high in the study area. Last child survival status, sex of last child, maternal age, contraceptive use, duration of breast feeding, and place of birth were determinant factors for short birth interval practice. Promoting institutional birth in collaboration with health extension workers, Creating awareness for parents through community based health education program to avoid child`s sex based birth interval practice and advice on advantage of using contraceptives while breast feeding to delay pregnancy might decrease short birth interval practice.

## Limitation of the study

Measuring birth interval with the women’s memory and duration of breast feeding might result recall bias. Though birth interval can be explained by social and cultural issues, the questionnaire did not extend to investigate such issue in depth.

## Abbreviations

AOR: Adjusted Odds Ratio
ANC: Antenatal Care
CI: confidence interval
DHS: Demographic and Health Survey
EDHS: Ethiopia Demographic and Health Survey
LNMP: Last Normal Menstrual Period
UOG: University of Gondar
USA: United States of America
WHO: World Health Organization
